# A biographical approach to studying individual change and continuity in walking and cycling over the life course

**DOI:** 10.1016/j.jth.2014.07.004

**Published:** 2014-09

**Authors:** Heather Jones, Kiron Chatterjee, Selena Gray

**Affiliations:** a Centre for Transport and Society, University of the West of England, Coldharbour Lane, Bristol BS16 1QY, UK; b Department of Health and Social Sciences, University of the West of England, Coldharbour lane, Bristol BS16 1QY, UK

**Keywords:** Walking, Cycling, Life course perspective, Biographical interviews, Typology

## Abstract

Most research studies seeking to understand walking and cycling behaviours have used cross-sectional data to explain inter-individual differences at a particular point in time. Investigations of individual walking and cycling over time are limited, despite the fact that insights on this could be valuable for informing policies to support life-long walking and cycling. The lack of existing longitudinal data, difficulties associated with its collection and scepticism towards retrospective methods as a means to reconstruct past behavioural developments have all contributed to this deficit in knowledge. This issue is heightened when the time frame extends to longer term periods, or the life course in its entirety. This paper proposes and details a retrospective qualitative methodology that was used to study individual change and stability in walking and cycling within a life course framework. Biographical interviews supported by a life history calendar were developed and conducted with two adult birth cohorts. Interpretive, visual biographies were produced from the interview materials. Analysis focused on identifying the occurrence, context and timing of behavioural change and stability over the life course. Typologies of behavioural development were generated to resolve common and distinct behavioural patterns over the life course. Whilst the validity of reconstructed biographies of walking and cycling cannot be proven, this is an approach which offers credible and confirmable insights on how these behaviours increase, diminish, persist, cease, are restored or adapted through the life course, and how behavioural trajectories of walking and cycling may be evolving through historical time.

## Highlights

• Individual change and stability in walking and cycling is poorly understood.
• Retrospective methods offer a life course perspective on these behaviours.
• We present a novel application of a biographical approach on the topic.
• Longitudinal typologies reveal common and distinct trajectories of walking and cycling.
• Findings can inform policy to support life-long walking and cycling.

## 1 Introduction

Recent studies have demonstrated health benefits of physical activity that are accrued in the long term from regular activity (Middleton et al., 2010, Pluijms et al., 2007, von Bonsdorff and Rantanen, 2011, Hirayama et al., 2010). Public health researchers have endorsed policies to promote walking and cycling over formal exercise as a more effective approach to tackle physical inactivity (Ogilvie et al., 2007). The predominant approach to research has been to examine the relationship between an individual’s prevailing propensity to walk and cycle and the contemporaneous physical, social and individual characteristics of the setting, and from this identify opportunities for promotion of walking and cycling. This conceptualises a static model of behaviour and its relationship to contextual factors. Research that reveals how individual behavioural outcomes have emerged through time may be more valuable for the development of interventions and policies that support life-long walking and cycling.

A much smaller body of research has concerned the dynamics (individual change and stability) of physical activity and travel behaviours. There is variation in the analytical focus, methodologies and temporal scope of this work. The complexity of measuring behaviours like walking and cycling and the challenge of following individuals over time are particular constraints in this area. Barnett et al. (2008) examined trajectories of individual behaviour constituted by prospective self-report of activity at three time points within a twenty-two year period (Barnett, et al., 2008). Latent class growth analysis identified distinct classes of trajectory and found that being female, older, having low income or lower educational achievement predicted a type of trajectory that was inactive or decreasing. Another seam of research has come about in both fields stimulated by the proposition that life events can induce behavioural change through disruption of the context for stable behaviours. Allender et al. (2008) reviewed prospective and retrospective studies of changes in physical activity levels occurring in the course of particular life events and transitions. Extending the temporal view to the whole life time qualitative retrospective methodologies have been used to explore sports participation (Tulle and Dorrer, 2012). Analysis here focused on changes in participation between stages of the life course, the meanings ascribed to physical activity in a retrospective view and how participation was shaped by the experience of ageing.

Travel behaviour researchers have looked at the association between certain life events and changes in travel behaviour revealed through prospective panel data (Dargay and Hanly, 2007) and retrospective self-completion questionnaires (Beige and Axhausen, 2008). The dynamics of walking and cycling are only revealed as far as their use as commute mode and the temporal span of panel surveys are periods far shorter than a life time. Further, whilst this reveals the propensity for the change to occur in the course of a life event but leaves opaque the process of change itself. Lanzendorf (2010) used retrospective qualitative reports of behaviour over a fifteen year period to understand the impact of entering parenthood on travel behaviour. This meant that the identification of a behavioural change, as well as the link between changes in life circumstances and the change in behaviour, originated with the individual.

Two studies have used qualitative retrospective data to look at trajectories of cycling behaviour. One found that behaviour changes in a three-year period were usually ascribed to life events (Chatterjee et al., 2013). Bonham and Wilson (2011) took a whole life view collecting complete histories of cycling from Australian women who had made returns to cycling. Social relationships were often implicated in returns made in the twenties, while health and fitness concerns were important for those in their thirties. Finally, Pooley et al. (2005)’s study of mobility histories provided a useful exemplar of making links between biographical data on individual mobility and social and structural changes.

In summary, walking and cycling has been only a minor concern in studies concerned with the dynamics of physical activity and travel behaviour. Second, there is very little study directed at behavioural development over longer term time frames; the greater body of dynamic studies look at behaviour change in the course of a particular event or transition, or in time periods of years to decades. Biographical methods offer a means to extend the temporal view on behaviour to the life course. Most informative for this study is Bonham and Wilson (2011)’s study of women’s cycling histories. However, their research covered only women who had made returns to cycling. It therefore does not offer insight on other outcomes, men’s experiences or development of walking.

Together this leaves much scope to develop understanding of how walking and cycling develops over time, specifically how they may increase, diminish, persist, cease, be restored or adapt and the longer term processes through which earlier behaviours evolve into later behavioural outcomes. For instance, socialisation (Haustein et al., 2009) and habituation (Bamberg et al., 2003) in the transport field, and genetic predisposition and habit formation in physical activity research (Telama, 2009) have been proposed as processes which shape behaviour over the longer term. Ambitiously then, it was the aim of this research to understand changes and continuities in individuals’ walking and cycling, as both transport and physical activity, at the temporal scale of the life course.

A life course perspective is a common theoretical orientation adopted in research into longer term individual development along various dimensions or domains of human lives and functioning. This is a set of concepts and principles that offer a longitudinal framework. It considers an individual “dynamically as the consequence of past experience and future expectation as well as the integration of individual motivation with external constraint” (Giele and Elder, 1998). The life course is conceptualised as a set of interwoven life-long developmental trajectories, embedded within an evolving historical context (Giele and Elder, 1998). Trajectories are viewed as non-linear patterns of forward movement that chart the course of the individual’s progression in a particular biological, psychological, behavioural or social dimension, being shaped by discrete changes and more gradual transitions and continuity (Li et al., 2009). Development is life-long and linked to and shaped by other lives in familial and social networks. The life course perspective has informed studies of spatial mobility (Kulu, 2008) and food choice (Devine et al., 1998, Delaney and McCarthy, 2009), but has not been explicitly adopted in studies of physical activity and travel behaviour. Adopting this conceptual framework then, an individual’s walking and cycling behaviour through life was conceptualised as a behavioural trajectory.

The absence of existing sources of data that could be mined for a whole life view of walking and cycling behaviour directed the study towards a retrospective method. Rather than tasking participants with giving a continuous report of levels of walking and cycling they were asked to describe and explain their behavioural change and continuity in the form of a life story. The use of narrative data oriented the study towards individual accounts which organised memories, perceptions and thoughts about walking and cycling behaviour into explanatory accounts which made explicit the connections between past behaviour and experiences and their current patterns of walking and cycling. This grounded insights on behavioural development in the experiences and perspectives of the individual, rather than ascribing connections between behaviour change and life events on the basis of them being contemporaneous

The strategy was to reveal using multiple individual accounts the occurrence and timing of developments in walking and cycling trajectories through the life course. Informed by the life course perspective a concern was also to explore whether walking and cycling trajectories were changing through historical time. The premise was that marked economic, technological, social and cultural changes in the UK in the second half of the twentieth century meant that birth cohorts separated by a few decades had encountered quite different physical, cultural, and social contexts for walking and cycling. One aim was to identify whether this was reflected in their behavioural trajectories. A further dimension was to examine whether within family groups trajectories were shaped by life events and transitions in linked life courses. It was hypothesised that events in the life of one generation could impact on the walking or cycling of a parent or child through an altering of the context for behaviour.

This paper introduces and explains the narrative biographical approach developed to study individual’s change and continuities in walking and cycling over the life course, an approach we believe has potential for wider usage in behavioural studies. The findings are published elsewhere (Jones, 2013). The following sections explain the method, data processing and analysis. The paper concludes with some critical reflection on the strengths and limitations of the approach.

## 2 Methodology

### 2.1 Life history interviews

**Fig. 1:**
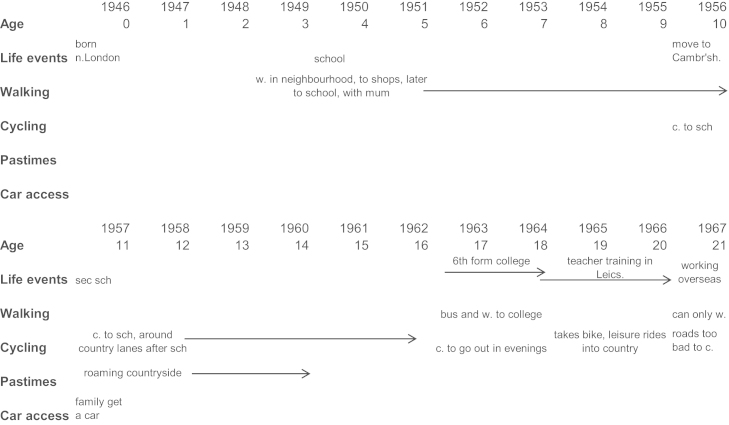
Life history calendar (LHC).

The biographical accounts were elicited in face to face interviews conducted over two occasions by the first author. The first interview opened with discussion of the participant’s current walking and cycling activity before they were asked to tell the interviewer about their walking and cycling over their life, describing the changes and stability in their behaviour as they saw them in relation to events and transitions in their life. Starting school, moving house, leaving home, getting married and having children were offered as examples of life events their story might entail. Whilst the participant spoke, the interviewer annotated a matrix known as a life history calendar (LHC) (see Fig. 1), identifying key events and recollections of regular, typical walking and cycling activity.

#### 2.1.1 Interview 1

The LHC was in view of the participant as it was completed. The structure of the LHC facilitated the retrieval of autobiographical memory, providing timing and substantive cues pertaining to life domains to structure the emerging life history information (Martyn and Belli, 2002). In this way the emerging life history supports the recall of less memorable past experiences and details (Martyn and Belli, 2002). LHCs have been used in the study of smoking histories (Parry et al., 1999), chronic health conditions (Dawson et al., 2003), employment histories (Fehring and Bessant, 2009) and family meals (Harrison et al., 2011).

If the participant stalled in their account the interviewer used the LHC to prompt them or probe their behaviour at that time with a question of the type “what sort of walking and cycling were you doing then?” or “Were there any changes to your walking and cycling at that time?”

The LHC was found to enhance the interviews in many ways. It was useful in initiating the interview and conveying the temporal focus of the study. It also engendered the collaborative nature of generating the eventual walking and cycling biography. With the LHC there was more even attention over the life course, thus overcoming a propensity noticed in pilot interviews for participants to explain at length their early life experiences and then condense their adult years. The format allowed the interviewer to capture both discrete events and behaviour change and more generalised recollections of behaviour where timing was less specific. This flexibility contrasts with the self-administered life history instruments of the sort used by Beige and Axhausen (2008) where the participant were directed by the structure to record pre-specified life events (such as birth of a child) and behaviour changes.

The LHC also accommodated recollections that emerged out of chronological order which meant that the participant was not confined to a strictly linear construction of their behavioural trajectory. The advantage of an interview situation was that the participant could be encouraged to greater depths of introspection and reflection. Finally, as well as eliciting a more coherent and complete life history, the graphical representation could make apparent aspects of the life history which might be contradictory or incoherent, allowing the interviewer to seek clarification thus improving the quality and accuracy of the data. Others have reported similarly (Parry et al., 1999, Dawson et al., 2003).

It was common for substantial periods of life to be covered with very little detail. The interviewer returned to these periods once the account was complete and probed for more detail. At the end of the first interview the participant was asked to give a reprise of their walking and cycling trajectories, separately. This allowed for a check on internal consistency and often gave an initial indication of the holistic dynamic of the behavioural trajectory.

#### 2.1.2 Interview 2

As far as possible interviews were conducted a week apart. In the interval the life history data was translated onto a personal timeline. Participants reviewed these at the start of the second interview and were asked to draw on the timeline to correct any misrepresentations. The intention was to converge the emergent representation with the participant’s understanding of their trajectory. Some offered further recollections in the second interview that were the result of reflection or research between interviews. These were added to the timeline. The interview then proceeded probing the circumstances of behaviour change and stability with questions composed in light of the first interview. The interviews lasted between thirty minutes and hour and a half with the second interviews being generally shorter.

Generally participants more readily described and explained the evolution of their cycling than their walking. Ownership or access to a bike provided a marker for potential cycling activity and periods of cycling and no cycling activity. Reasons for continuity, returns to and departures from cycling seemed to be more easily recalled and articulated. Walking trajectories, in contrast, required more questioning from the interviewer, questions that probed whether and how walking had changed over particular transitions and events.

### 2.2 Study participants

The aims of the research were best served by a purposive sample. Parent-child dyads were recruited where the parent was born between 1945 and 1955 and the (adult) child between 1975 and 1985. This gave insight into trajectories at earlier and later stages of adulthood and that intersected differently with historical time. The sample was also constructed to comprise some variation in life course circumstances and walking and cycling outcomes, the rationale being that this would translate to a broader range of walking and cycling experiences and thereby extend the breadth of insight offered on trajectories in general. To this end the study group was selected to be gender-balanced with variation in level of education, occupational background, the degree of urbanity of residential area experienced in childhood and adulthood as well as current engagement in walking and cycling.

Sample size was a trade-off between what was a manageable body of data to work with and the number and distribution of cases that would render a broad insight into the phenomena. Recruitment was conducted in series so that later participants were recruited with reference to those already included. An initial target of five participants in each cohort-gender group was exceeded to include participants who served to broaden the sample. In total there were thirty-three participants; eight males and eight females in the older cohort and eight males and nine females in the younger cohort. This provided twenty-one dyads across ten separate family groups.

Recruitment was conducted through community groups in Bristol, a city in the south west of England, and through the personal networks of the researcher and the participants themselves. One member of each dyad was resident in the greater Bristol area. All participants gave written consent to take part in the study which was granted ethical approval by the University of the West of England’s Research Ethics Committee.

### 2.3 Data processing and analysis

The aim of analysis was to engage with the diachronic dimension of the data and preserve the integrity of the when’s, how’s and why’s of each account to the explanatory narrative of current behaviour. This was an approach consistent with holistic content analysis in Lieblich et al. (1998)’s classification of narrative analysis. This diverged from more traditional approaches in qualitative research where there is abstraction of data units to identify themes across cases.

**Fig. 2:**
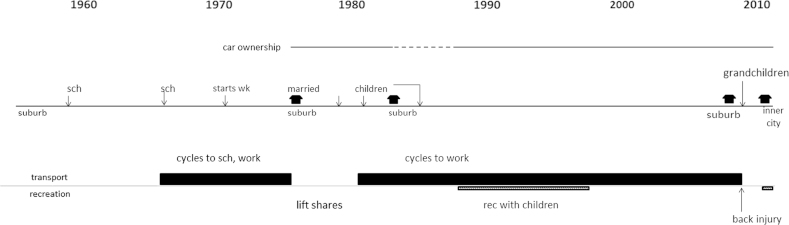
Visual biography.

**Fig. 3:**
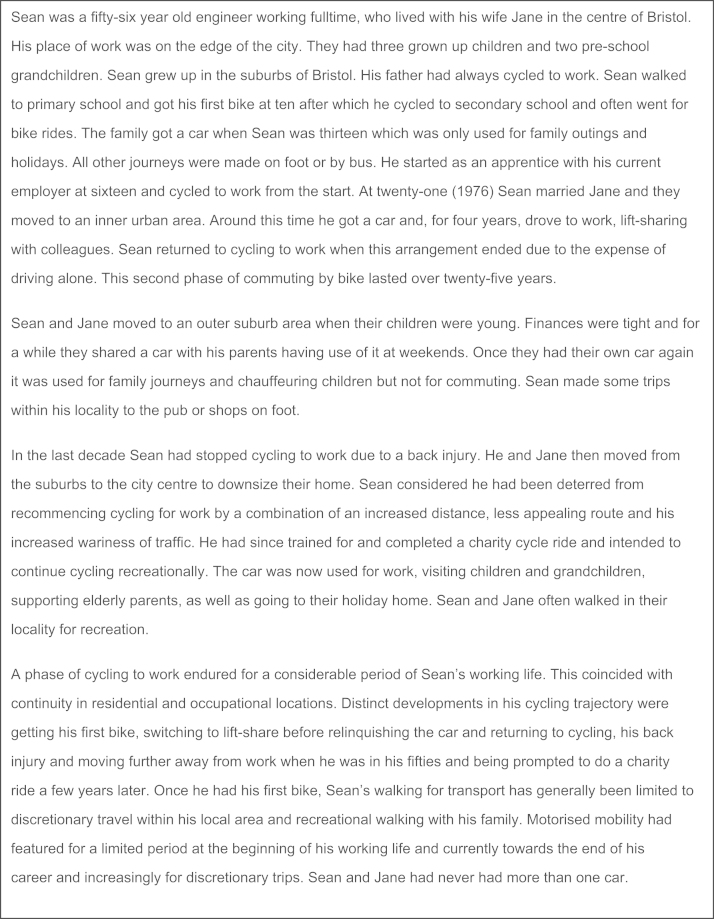
Text biography.

Data processing and analysis were conducted by the interviewer (first author). The first step involved distilling and interpreting a chronological account of events and behaviour developments from the large volume of data generated for each case (interview recordings, LHC and timeline). A visual schematic and a descriptive and interpretive text were developed in parallel through repeated listening to the interview recordings and study of the LHC. Fig. 2, Fig. 3 present examples of the visual and an abridged text biography.

**Fig. 4:**
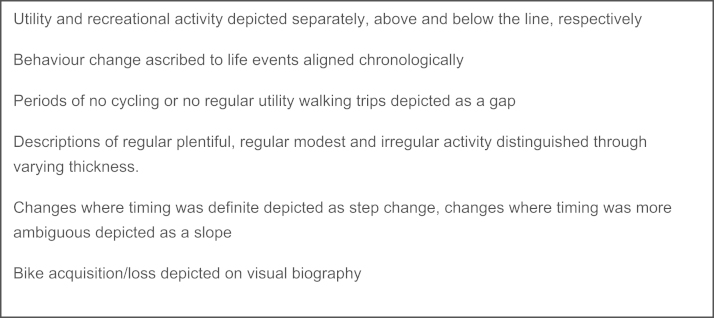
Decision rules for drawing visual biography.

The visual was developed from the personal timeline with the addition of a parallel line, hand-drawn according to a set of decision rules (Fig. 4). Confirmability of producing these visual biographies was explored by having research supervisors replicate the process from interview transcripts for a couple of cases. Overall corroboration with the version produced by the lead researcher was found to be good.

The text component started as a detailed summary of the participants’ account, working directly from the audio and the timeline. This incorporated the details participants had given to contextualise past behaviour and behavioural changes. Temporal ordering of the text initially mirrored the progression of the interview and some verbatim quotes were included. This was then condensed and partially reordered to reflect the biographical order of the participant’s life history and clarify the narrative linkages that were made between events and changes and continuities in behaviour. Finally, the text was then prefaced with a descriptive introduction of the participant and their current situation, and ended with some interpretive statements that took a holistic perspective on the dynamics of the trajectory. Dyad biographies were produced by presenting the schematic the child’s trajectory below that of the parent and drawing vertical lines to connect linked life events. This allowed trajectory developments to be viewed in the context of their linked life course.

The visual and text biographies transformed the data from the rich portrayals of individual experience generated in the interview to a format amenable to comparative analysis of cases, cohorts and dyads. It also helped the researcher communicate and discuss the cases with wider audiences. The process of producing these enhanced the researcher’s familiarity and understanding of the particularities of each case, and generated some preliminary observations on gender and cohort differences.

Analysis progressed in a data-led manner with the visual examination of individual cases in cohort and gender-cohort groups and parent-child dyads, focusing on the timing and circumstances of change and continuity. Throughout analysis reference was made to the text biographies as needed. Preliminary observations were revisited to assess their applicability across all cases. Transition to adulthood and, more tentatively, later working life were identified as a period when life events could occur that changed opportunities and constraints for active travel. This prompted examination and comparison of cases in terms of their situation regarding education, employment, family and mobility resources and travel behaviours through these periods.

**Fig. 5:**
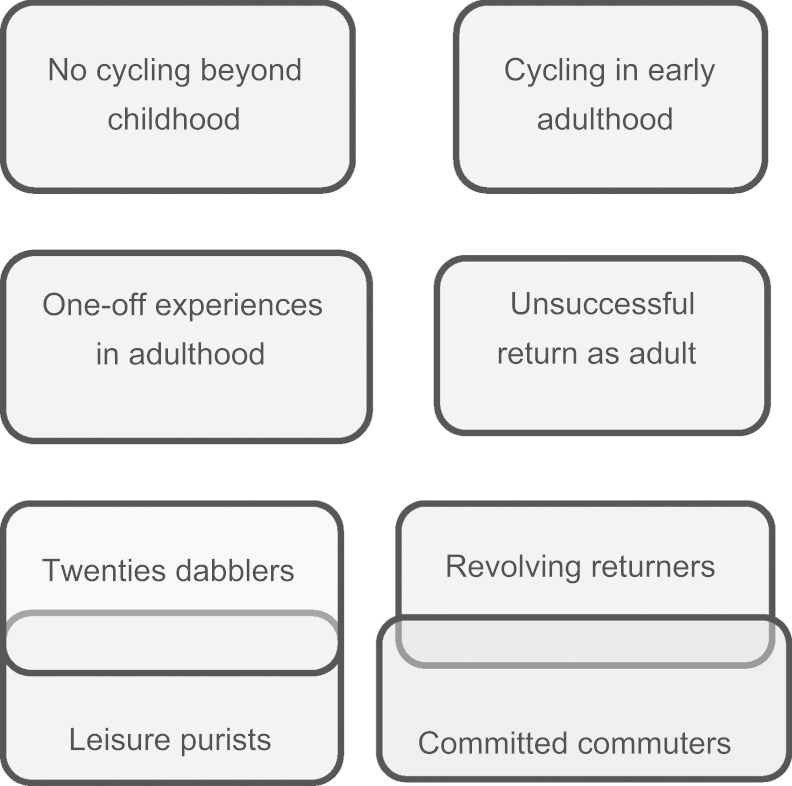
Typology of cycling trajectories.

**Fig. 6:**
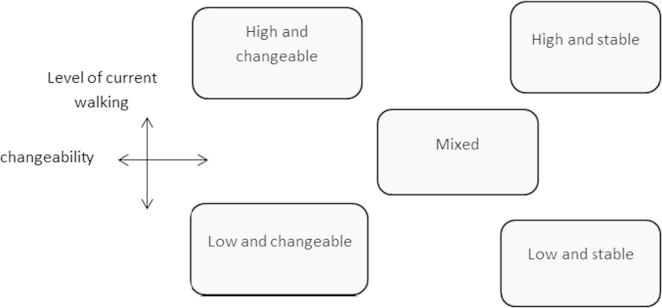
Typology of walking trajectories.

A further step was categorisation of trajectories by type, separately for walking and cycling based on the researcher’s assessment of similarities and differences. The resultant typologies are depicted in Fig. 5, Fig. 6. Groups more readily emerged for cycling trajectories than walking trajectories. The cycling typology also reflected different types of cycling, e.g. recreational, transportation or mixed. The first groups were those trajectories which demonstrated more stability, i.e. those with fewer separate phases of cycling or non-cycling. Thereafter cases were assigned to existing groups or new groups, according to similarity and difference. Preliminary groupings were finalised through an iterative consideration of within-group homogeneity and between-group heterogeneity. Some groups were merged or overlapped, resulting in eight groups.

For walking, in the absence of clearly distinguishable groups, groupings were established by positioning of cases on a matrix according to level of change over the trajectory and current level of regular walking for transport (low, moderate or high). A prototype illustrating degrees of changeability was developed as a reference for positioning cases. More moderate cases were positioned with reference to extreme ones and preliminary positions were finalised through iterative consideration of relative positioning. Five groupings were identified from the clustering of the cases and named according to their positioning (e.g. low and changeable).

These typologies are presented here for illustration of the method—explanation and interpretation of their implications for policy is available elsewhere (Jones, 2013).

Ongoing discussion was had with research supervisors on analytic steps, interpretation and emergent findings from the data as analysis advanced. At a fairly advanced stage a workshop was held with practitioners who had a role in the promotion of walking and cycling. This was a further occasion to establish and enhance the trustworthiness of the research. More specifically, the purpose was to test the plausibility and relevance of the findings with a practitioner panel.

The workshop confirmed the currency of life transitions in practice as a target for intervention to change behaviour. However, less discussion was generated on how policy and practice might respond at a population level to in-depth understandings of individual behavioural trajectories and typologies of such trajectories. In light of the workshop the concluding stages of analysis took a heightened focus on translation of knowledge for praxis.

Analysis culminated in a written description and explanation of common and distinct behaviour changes in the context of life transitions through the life course and the typologies. A framework was developed, drawing on life course research on food choice trajectories (Delaney and McCarthy, 2009), that conceptualised individual life course trajectories of walking and cycling. This provided a platform for thinking of trajectories as embedded within evolving micro and macro contexts which present opportunities and constraints for walking and cycling over the life course. This synthesis drew upon literature which offered historical perspectives on the impact of social, structural and spatial changes in the UK over the twentieth century on travel behaviour (Pooley et al., 2005, Chatterjee and Dudley, 2008). Central to this was a book written specifically to provide social and historical context for the lives charted in the British birth cohort studies (Wadsworth and Bynner, 2011).

## 3 Discussion and conclusion

This paper has outlined a methodology that advances the study of walking and cycling behaviour over time from limited time periods towards a whole life view. Biographies of walking and cycling require a broader view of what constitutes knowledge than has underpinned the main body of research on travel behaviour and physical activity. The approach served the research objective of examining the dynamics of walking and cycling through the life course, a temporal scale that is generally unparalleled in the extant literature. The remainder of this article is given over to reflection on the strengths and limitations of the research design and their implications for the contribution to knowledge.

It demonstrates good research practice to consider how knowledge is constituted by research methods. The biographies were not considered to provide an unadulterated view of individual’s walking and cycling, being mediated through human memory and communication. Participants’ behaviour through time was considered to exist in reality, independently of attempts to have knowledge of it. The trajectory was a conceptual tool presented to the participant for communication, and used by the researcher to apprehend, interpret and compare across cases. The trajectory was a collaborative construction between participant and researcher that was rendered, after the interviews, in the form of the biography.

The interview, handling and analysis of the data, were taken to be shaped by the current social norms around physical activity and travel behaviour and indeed cultural conventions on the telling of life stories. It was likely that participants were inclined to provide a more socially desirable account of their behaviour. To empower participants to report behaviour patterns that might contravene values around healthy and environmentally responsible behaviour the researcher emphasized the value of understanding life histories where walking and cycling had been less prominent. It was not presumed that the influence of social norms and values was nullified by this but that it enabled a broader range of trajectories to be acquired.

In telling a life story people select from their autobiographical memory. The teller is obliged, in order to present an account that is coherent, to resolve past changes and continuities into the current behavioural state. It is considered then that the methodology was oriented to changes in walking and cycling which were more easily remembered and instrumental to the overall trajectory. Participants were inclined to compose a story around a few pivotal moments, rather than a series of incremental or apparently inconsequential changes. This reduction was countered to a certain extent by the structuring of the account by the LHC and the opportunity for the interviewer to return to certain time points in the course of the interview. However, it is surmised that the methodology may have imposed an overly rationalised, linear form on these biographies and was prone to leave uncovered instances of less significant and impermanent change.

These are a few issues that complicate the view of insights from biographical studies. However many of these issues are not exclusive to retrospective, qualitative data but are common to inquiry that seeks to understand complex human actions through individual׳s report of those actions. Neither is it essentially problematic. Some level of reduction was necessary to make the trajectories tangible and amenable to comparative analysis and discussion. Accepting this rationalisation, this revealed those developments salient to the participants in their understanding of how their current behaviour patterns had arisen.

Had it been possible to use prospective data, participants would have been closer in time to the experiences and behaviours they were reporting. The frame of reference at each occasion, however, would have been different. Instead, past experiences were reported with awareness of present outcomes and in light of the intervening experience. This does not diminish authenticity. Indeed, it permitted more enlightened reasoning of past changes, so that participants could reflect on the significance of a change for the trajectory, and the extent to which it was precipitated by contemporaneous factors, preceding events or indeed future orientations.

Analysis here was directed towards the content of the biographies and variation across cases. An alternative direction would have been to engage with how life stories of walking and cycling were constructed, exploring the use of language, the meanings deployed, the collaboration between narrator and listener and possible identities constructed. Certainly, common narrative structures were recognised in the data. We anticipate this reflects the impression of cultural values and norms and conventions of telling on life stories of walking and cycling. Approaching the data this way could be enlightening on matters of intrinsic attitudes, motivations and concepts of self that shape current behaviour and expectations of future behaviour and help us understand how the cultural context may prescribe certain forms of walking and cycling trajectory. Indeed some scholars of narratology assert that narrative resources shape lives in a ‘predisposed, tacit embodied way’ (Frank, 2010).

To our knowledge, the generation of longitudinal, qualitative typologies makes a novel methodological and substantive contribution to the field. In putting forward such a typology it is important to recognise its political dimension—its potential as a mode of classification to censure individuals for following seemingly sub-optimal or detrimental trajectories. We see the typologies as heuristics to comprehend and harness the explanatory power of biographies and consider it possible for them to be utilised in a way that is not detrimental to individuals. Although not detailed in this paper, the typologies are an attempt to illuminate the evolution of opportunity structures for walking and cycling and how certain behavioural outcomes arise in the succession of common life events and transitions. Attention to social and spatial factors which impose constraints offers a basis to develop interventions to support life-long walking and cycling. We would advocate that the translation of such knowledge be oriented to the socio-spatial constraints of walking and cycling, in the context of individual factors, to limit the potential for interventions which stigmatize individuals by their choices.

Integration of the biographical approach and life course perspective with other methodologies is required to fully realise its potential to contribute to the field. A suggested way forward is to test hypotheses formulated on biographical insights in panel surveys or cohort studies. Such analyses require the inclusion of questions in multi-purpose longitudinal questionnaires that give a fuller picture of walking and cycling activity beyond their use as commute mode.

In conclusion, biographical interviews and life history calendars have featured previously in physical activity and travel behaviour research. The novelty here lies in their combination to study walking and cycling at the scale of the life course. Further innovation is offered in the development of a grouping process to generate longitudinal typologies of trajectories that offers a platform for both research and practice.
